# Winner and losers: examining biotic interactions in forbs and grasses in response to changes in water and temperature in a semi-arid grassland

**DOI:** 10.1093/aobpla/plad017

**Published:** 2023-04-24

**Authors:** Morodoluwa Akin-Fajiye, Laura W Ploughe, Amber Greenall, Lauchlan H Fraser

**Affiliations:** Department of Natural Resource Science, 805 TRU Way, Thompson Rivers University, Kamloops, BC, Canada V2C 0C8; Department of Natural Resource Science, 805 TRU Way, Thompson Rivers University, Kamloops, BC, Canada V2C 0C8; Department of Natural Resource Science, 805 TRU Way, Thompson Rivers University, Kamloops, BC, Canada V2C 0C8; Department of Natural Resource Science, 805 TRU Way, Thompson Rivers University, Kamloops, BC, Canada V2C 0C8

**Keywords:** Climate change, exotic, forbs, grasses, native, warming, water

## Abstract

Warming and changing water amount can alter the outcome of biotic interactions in native and exotic plants between facilitation and competition. Exotic plants may adapt better to changing environmental conditions, such that they may compete better than native plants. We conducted competition trials for four plant species, two exotic forbs (*Centaurea stoebe* and *Linaria vulgaris*) and two grasses (exotic *Poa compressa* and native *Pseudoroegneria spicata*), commonly found in Southern interior British Columbia. We compared the effects of warming and changing water on target plant shoot and root biomass, and on pair-wise competitive interactions among all four species. We quantified interactions using the Relative Interaction Intensity index, which has values from −1 (complete competition) to +1 (complete facilitation).

*C. stoebe* biomass was highest under low water and no competition. Facilitation of *C. stoebe* was found under high water and low temperatures but shifted to competition under low water and/or warming. Competition in *L. vulgaris* decreased due to reduced water and increased due to warming. Grasses were less competitively suppressed by warming but more competitively suppressed by reduced water input. The response of exotic plants to climate change can differ by plant species, moving in opposite directions for both forbs, but grasses appear to respond similarly. This has consequences for grasses and exotic plants in semi-arid grasslands.

## Teaser

As climate change continues to be of worldwide concern, interactions between plant functional groups such as grasses and forbs, or native and exotic species are expected to change as plants response to changes in water content and warming. In this greenhouse study, response to elements of climate change was individual, rather than based on plant functional groups. Further studies in field settings, extended to a wider range of competitive interactions may help elucidate the impact of competition on plant species in grasslands.

## Introduction

Global climate change has implications for the structure and functioning of many ecosystems. Climate change has modified precipitation patterns and warming leading to loss of important ecosystems and plant communities (Karl *et al*. 2009; Zuo *et al*. 2020). The transport of exotic species across geographic boundaries, and their ability to alter above and below ground communities in the new location, are well documented (Kourtev *et al*. 2002; Levine *et al*. 2003) and is another major threat to native biodiversity (Dawson *et al*. 2012). Both components of global change can interact synergistically to the disadvantage of native species (Liu *et al*. 2017; Giejsztowt *et al*. 2020). Consequently, to inform mitigation policies surrounding native biodiversity loss and invasive species, it important to understand how relationships between native and invasive plants might be altered due to different drivers of global change.

Many ecosystems are predicted to experience accelerating temperature changes over the next 100 years, reflecting the need to understand how plant communities will respond (Loarie *et al*. 2009). Shifts in biotic interactions, such as competition and facilitation, between native and exotic species may result from changes in components of climate change (Suttle *et al*. 2007; Angert *et al*. 2013). In addition, in highly diverse dryland communities in which water is a limiting resource, the ecosystem is highly dependent on consistency in water availability, and small changes in the availability of water can have substantial effects (Tietjen *et al*. 2017). Similarly, altered rainfall regimes can directly, and indirectly via changes in soil moisture, influence plant aboveground biomass, and competitive interactions (Lenihan *et al*. 2003; Ploughe *et al*. 2019). In British Columbia, climate change is expected to result in warmer temperatures, higher annual precipitation, lower summer precipitation, and longer summer droughts (Gifford *et al*. 2022). Invasive species may adapt to these changing conditions by displaying greater phenotypic plasticity or may be competitively superior to native species due to a lack of natural enemies (Ferrenberg *et al*. 2018; Huang *et al*. 2020). Thus, different species of native and exotic plants may display unique responses to changing conditions, allowing climate change to generate winners and losers in different parts of the world (Buckley and Csergő 2017).

Biotic interactions such as facilitation and competition may mediate the effects of climate change on individual species (HilleRisLambers *et al*. 2013; Srivastava *et al*. 2021). For example, Sheley and James (2014) found that drought stress led to weaker competitive effects of an invasive annual grass on a native perennial grass than vice versa. Alba *et al*. (2019) showed that in the presence of drought, facilitation of native longleaf pine seedlings by invasive cogongrass in early invasion stages, shifted to competition in the later stages of invasion. Giejsztowt *et al*. (2020) found warming increased the intensity of competition by an invasive plant indicating that climate change and plant invasions can combine to suppress native species.

In this study, we used pair-wise experiments (Keddy and Shipley 1989) to understand the nature of biotic interactions in native and exotic plants grown in pairs under simulated climatic conditions. We used the relative interaction intensity, which has limits of −1 to +1, in which positive values represent facilitation and negative values, competition, as an index of biotic interaction (Armas *et al*. 2004). We compared the responses of a native grass: bluebunch wheatgrass (*Pseudoroegneria spicata* Löve), an exotic forage grass: Canada bluegrass (*Poa compressa*), two exotic forbs: spotted knapweed (*Centaurea stoebe*), and yellow toadflax (*Linaria vulgaris*). Bluebunch wheatgrass is considered an economically important forage grass for livestock and wildlife grazing on western rangelands (van Ryswyk *et al*. 1966; Quinton *et al*. 1982). Canada bluegrass is native to Eurasia, but has become naturalized, and is now an important forage grass in North America (Oakley 1910; Stewart and Hebda 2000). Spotted knapweed is one of the most problematic invaders of British Columbian grasslands (Gayton and Miller 2012). Spotted knapweed can thrive under dry conditions (Lacey *et al*. 1989), although studies have indicated reductions in knapweed density and performance due to drought alone (Boggs and Story 1987), drought combined with competition with native grasses (Pearson *et al*. 2017), or competition combined with nutrient availability (Knochel *et al*. 2010). Yellow toadflax is native to Europe and was one of the first documented invasive plants in North America (Mack 2003). Yellow toadflax prefers relatively wet riparian environments and gullies and possesses a deep tap root that can compete for water and nutrients (Sing *et al*. 2016). It can spread via seed or shoot production from creeping rhizomes (McClay and De Clerck-Floate 2001; Ward *et al*. 2009). While yellow toadflax is not as widely studied or as widely distributed as spotted knapweed, it is present in southern British Columbia (McClay and De Clerck-Floate 2001) and is designated by the Forest and Range Practices Act as an invasive plant that should be monitored and controlled (B.C. Reg. 18/2004). Both exotic forbs can be found in a variety of habitats ranging from moist to dry soils in rangelands and along disturbed roadsides (Harris and Cranston 1979; Pauchard *et al*. 2003; Klinkenberg 2021).

The four plant species were tested under two temperature and two water regimes in a greenhouse pair-wise competition trial. We addressed the question: how do reduced water inputs and elevated temperature combine to alter performance and biotic interactions of native and exotic grasses and exotic forbs during plant establishment? We expect that more stressful experimental conditions will lead to weaker competitive interactions or facilitative interactions for target exotic forbs, and stronger competitive interactions for target native and exotic grasses. We expect that the forbs will be less competitively suppressed (i.e. display less negative Relative Interaction Intensity index, RII) compared to the grasses. We used early establishment of grasses and forbs to test these predictions, as early establishment success can confer an advantage to growing plants (Donohue *et al*. 2010).

## Methods

### Experimental design

The experiment was conducted between January and March 2009 at the Thompson Rivers University Greenhouse. We planted four species such that each species was in competition with itself and with each of the other three species. In addition, each plant was planted alone without any competitor. Two were exotic forbs (spotted knapweed and yellow toadflax), while two were native and exotic grasses (bluebunch wheatgrass and Canada bluegrass, respectively). Seeds from multiple individuals of each species were collected in Fall 2008 in Kamloops, British Columbia and mixed to ensure genetic variation. Seeds were stored at ~25 °C for approximately three months and then germinated on a medium of sterilized sand and distilled water. Upon germination, individuals were allowed to grow until they attained a root length of between 5 and 15 mm, before the fully elongated cotyledons were potted. Seeds were transplanted into pots, which were 12 cm high and 8 cm diameter. Individuals in the same pot were transplanted on the same day.

The experiment included three two-level treatments that manipulated competition, temperature, and water inputs, respectively. For the competition treatment, each species was grown alone, i.e. one plant per pot, or in competition with another plant in one of four pair-wise combinations with either itself or one of the other three species. This amounted to *n* = 14 different pot compositions (*n* = 4 with one plant and *n* = 10 pair-wise combinations). Each pot composition was grown under four different environmental contexts determined by temperature and water treatments described below for a total of 56 treatment combinations. Ten replicates were included per treatment combination for a total of 560 pots. Plants were randomly assigned to treatments.

The greenhouse contained four independent pods—two were at low temperatures (25 °C/22 °C, day/night) and two were at high temperatures (27 °C/25 °C, day/night). The low treatment reflects current average growing season temperatures in the southern interior of (Environment Canada 2022), while the elevated temperature treatment was used to simulate a predicted 2 °C increase in temperatures by 2060, due to climate change (Masson-Delmotte *et al*. 2021). Each pot received either ‘high-’ or ‘low-’ water inputs. The high-water treatment received a weekly addition of 250 mL of Rorison’s solution (a nutrient solution composed of Ca/N, Mg, K/P, Fe, and trace elements such as Mn, B, Mo, Zn and Cu, see Hendry and Grime (1993)) and an additional 100 ml of distilled water halfway through the week, and the low-water treatment received solely the weekly addition of Rorison’s solution. The experiment ran for twelve weeks in the controlled greenhouse environment. Plants received 8 h of darkness and 16 h of light with 65 % day humidity and 80 % night humidity. During the experiment, soil moisture was measured such that percent volumetric water content (VWC) was around 20 % for high water and 12 % for low water. A previous study showed that VWC in this region is around 20 % (McCulloch 2013). At the end of the 12th week of planting, all individuals in the 560 pots were removed from sand, separated to roots and shoots (shoots were above the sand level in the pots, while roots were below the sand level) and oven-dried at 65 °C for a minimum of 48 h using a constant temperature oven (Yamato DKN812). When harvested, plants were still at the vegetative stage and had not begun to produce inflorescence. Dried root and shoot biomass were separately weighed using an analytical balance (Fisher-Scientific accu-225D).

### Data analysis

We tested the effects of water inputs (low vs. high), temperature (low vs. high), and competition (present vs. absent) on log-transformed shoot biomass and root biomass of target plants using four linear mixed models (LMMs), one for each target plant. We used pot and pod as random factors in our model to account for correlated error structures that may arise from plants sharing the same pot or pod. For each pot with two plants, one plant was designated as a target plant, while the second was designated as the competitor. Although we constructed individual models for each target plant, our experimental design was such that all four plant species experienced competition with one another, allowing for comparisons between all species.

To understand how plant interactions varied with temperature and water input, we calculated the RII for each target plant. The RII compares the biomass of plants grown alone to the biomass when target plants are grown with competitor plants, to understand the effects of competitor plants on target plants (Armas *et al*. 2004). RII is calculated using the formula: $RII=\;B_{w}-\;B_{0}/B_{w}+\;B_{0}$, where $B_{w}\;$ is the biomass of a target plant growing with a competitor plant, while $B_{0}$ is the biomass of the target plant grown alone. The RII has limits of −1 (complete competition) to +1 (complete facilitation) (Armas *et al*. 2004). RII allows us to consider proportional changes in biomass attributable to competitors under differing treatment scenarios. For each target plant, we tested the impact of RII on temperature (low vs. high), water input (low vs. high), and competitor identity using LMMs, constructed for each of the four species.

We interpreted RII of a target plant as the response of the target plant to the competitor plant under the given experimental condition. A significant competitor effect indicates that the target plant RII in response to competitors depends on the identity of the competitor plant. A significant water or temperature effect indicates that changes in either factor can affect the intensity of competition or facilitation for a given target plant. We calculated two RII values: one using shoot biomass, and another using root biomass to identify if competitive or facilitative responses of target plants differed between shoots and roots. We used maximum likelihood estimation within the lme4 package and compared models with and without the pod random factor to identify the model with the lower Akaike information criterion (AIC). Models including the pod random factor generally had lower AICs, for biomass, while those excluding the pod random factor had lower AICs for RII. For biomass and RII, models with lower AICs were used to obtain Type III ANOVA tables with Satterthwaite’s method (Bates *et al*. 2007). We conducted all analyses in R version 3.5.2 (R Core Team 2019), using the lme4 package (Bates *et al*. 2007). We used the ‘emmeans’ package to conduct *post hoc* tests, after adjusting for multiple comparisons (Lenth *et al*. 2018).

## Results

### Shoot and root biomass

**Figure 1.**
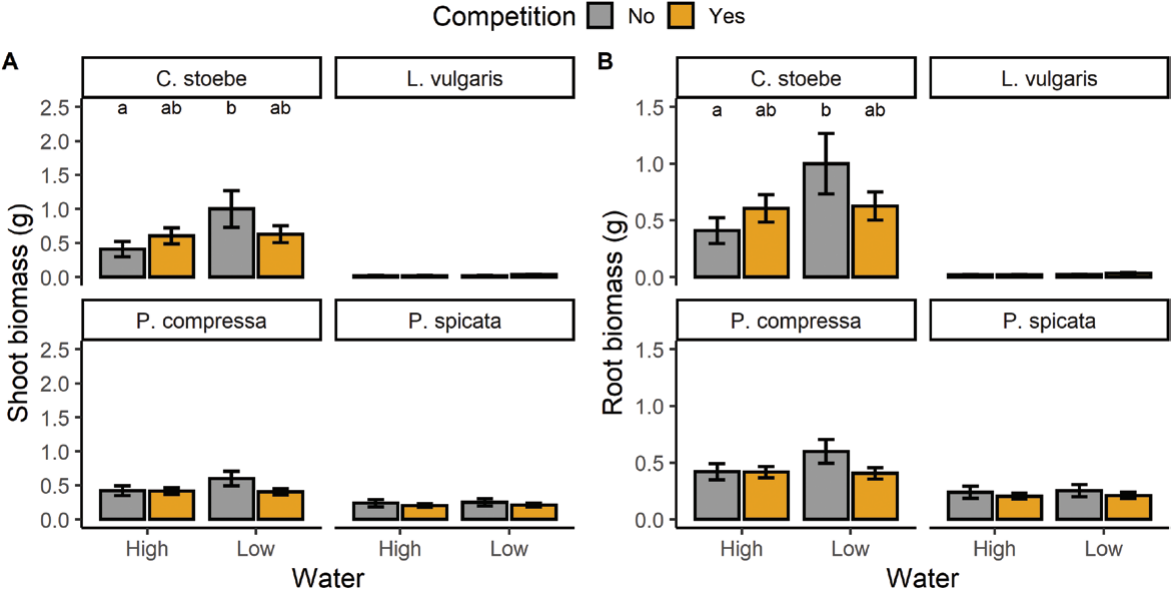
Mean ± 1 SE of (**A**) shoot biomass and (**B**) root biomass of the four plants in this experiment in response to water input and competition. Bars sharing the same letter are not significantly different at *P* < 0.05. Bars without letters show no significant interaction effects between water and competition.

For spotted knapweed, low water input led to higher biomass when there was no competition but did not influence biomass in the presence of competition*. Post hoc* tests showed that shoot and root biomass of spotted knapweed performed best under low water and no competition (Fig. 1A and B, Tables 1 and 2). Yellow toadflax showed only a significant main effect of water for shoot biomass (Table 1), in which plants with low water input had a higher shoot biomass than those with high-water input **[see****Supporting Information Table S1****]**. Bluebunch wheatgrass shoot and root biomass were suppressed by elevated temperatures **[see****Supporting Information Table S2****]**. Canada bluegrass and bluebunch wheatgrass displayed lower shoot biomass in the presence of competition but were unaffected by water or temperature (Table 1, [**see****Supporting Information Tables S3 and S4**]).

**Table 1.** Three-way ANOVA table testing the relationships between water, temperature, and the presence of competitors on shoot biomass of two invasive forbs (spotted knapweed: *C. stoebe* and yellow toadflax: *L. vulgaris*) and two forage grasses (Canada bluegrass: *P. compressa* and bluebunch wheatgrass: *P. spicata*). Values in bold are significant at *P* < 0.05.

	Centaurea stoebe			Linaria vulgaris			Poa compressa			Pseudoroegneria spicata		
	df	*F*	*P*	df	*F*	*P*	df	*F*	*P*	df	*F*	*P*
Temperature	1, 7	0.09	0.77	1, 6	1.87	0.23	1, 5	3.01	0.15	**1, 12**	**9.40**	**0.01**
Water	**1, 221**	**7.71**	**0.01**	**1, 208**	**4.52**	**0.03**	1, 228	2.13	0.15	1, 226	0.32	0.57
Competition (present/Absent)	1, 221	1.47	0.23	1, 208	0.19	0.67	**1, 228**	**15.40**	**< 0.01**	**1, 226**	**9.03**	**< 0.01**
Temperature × water	**1, 221**	**4.68**	**0.03**	1, 208	2.69	0.10	1, 228	2.59	0.11	1, 226	0.23	0.63
Temperature × competition	1, 221	1.30	0.25	1, 208	0.02	0.90	1, 228	0.91	0.34	1, 226	2.21	0.14
Water × competition	**1, 221**	**8.47**	**<0.01**	1, 208	1.40	0.24	1, 228	1.82	0.18	1, 226	1.55	0.22
Temperature × water × competition	1, 221	2.42	0.12	1, 208	0.11	0.74	1, 228	3.23	0.07	1, 226	0.71	0.40

**Table 2.** Three-way ANOVA table testing the relationships between water, temperature, and the presence of competitors on root biomass of two forbs (spotted knapweed: *C. stoebe* and yellow toadflax: *L. vulgaris*) and two grasses (Canada bluegrass: *P. compressa* and bluebunch wheatgrass: *P. spicata*). Values in bold are significant at *P* < 0.05.

	*Centaurea stoebe*			Linaria vulgaris			Poa compressa			Pseudoroegneria spicata		
	df	*F*	*P*	df	*F*	*P*	df	*F*	*P*	df	*F*	*P*
Temperature	1, 6	0.00	0.99	1, 6	4.75	0.07	1, 6	6.05	0.05	**1, 9**	**7.87**	**0.02**
Water	**1, 221**	**8.43**	**< 0.01**	1, 206	3.30	0.07	1, 208	2.33	0.13	1, 216	0.06	0.80
Competition (present/absent)	1, 221	0.05	0.82	1, 206	1.88	0.17	1, 208	3.58	0.06	1, 216	1.12	0.29
Temperature × water	**1, 221**	**5.18**	**0.02**	1, 206	2.98	0.09	1, 208	0.07	0.78	1, 216	1.22	0.27
Temperature × competition	1, 221	3.17	0.08	1, 206	0.00	0.98	1, 208	0.54	0.46	1, 216	3.10	0.08
Water × competition	**1, 221**	**7.17**	**0.01**	1, 206	1.20	0.28	1, 208	3.09	0.08	1, 216	0.00	0.95
Temperature × water competition	1, 221	3.30	0.07	1, 206	0.01	0.92	1, 208	0.37	0.55	1, 216	0.49	0.48

### Relative interaction intensity

**Figure 2.**
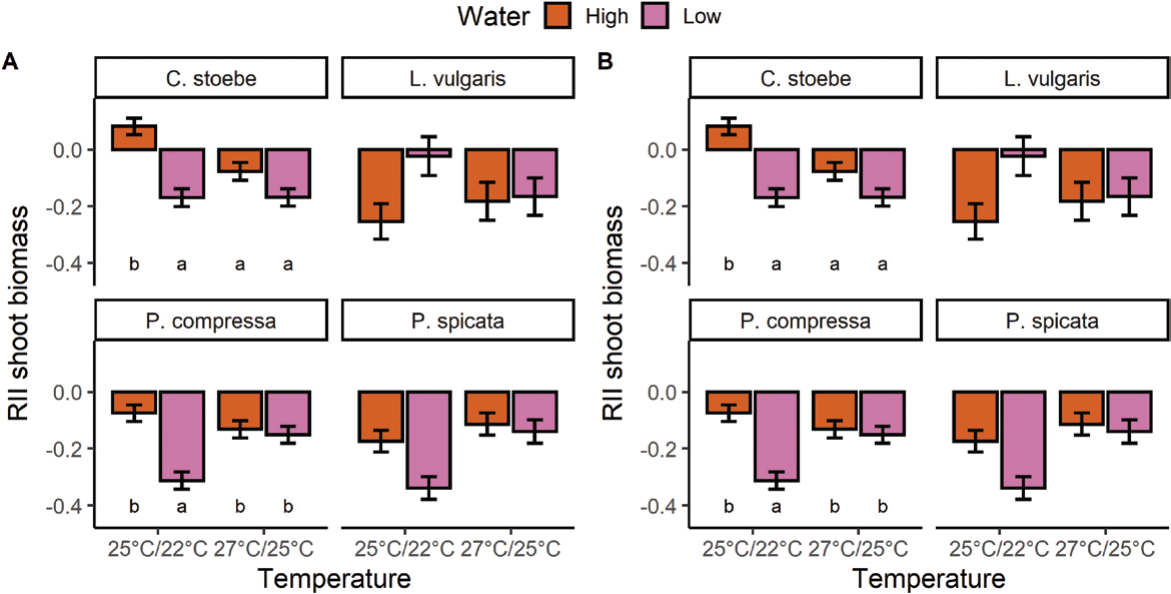
Mean ± 1 SE of relative interaction indices (RII) calculated using (**A**) shoot biomass and (**B**) root biomass of spotted knapweed (*C. stoebe)* and Canada bluegrass (*P. compressa)* in response to changes in water input and temperature. Bars sharing the same letter are not significantly different at *P* < 0.05. Bars without letters show no significant interaction effects between water and temperature.

**Figure 3.**
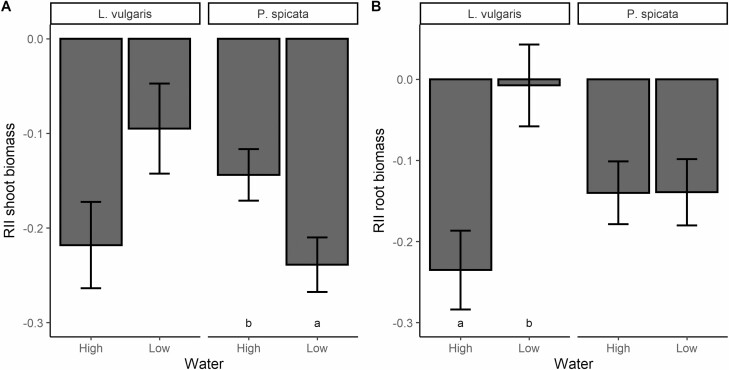
Mean ± 1 SE of relative interaction indices (RII) calculated using (**A**) shoot biomass and (**B**) root biomass of yellow toadflax (*L. vulgaris)* and bluebunch wheatgrass (*P. spicata)* in response to changes in water input. Bars sharing the same letter are not significantly different at *P* < 0.05. Bars without letters show no significant effects of water input.

**Figure 4.**
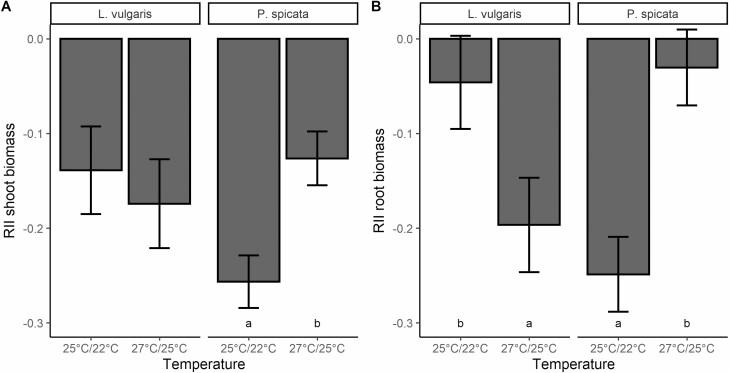
Mean ± 1 SE of relative interaction indices (RII) calculated using (**A**) shoot biomass and (**B**) root biomass of yellow toadflax (*L. vulgaris*) and bluebunch wheatgrass (*P. spicata*) in response to changes in temperature. Bars sharing the same letter are not significantly different at *P* < 0.05. Bars without letters show no significant effects of temperature.

Both shoot and root RII of spotted knapweed and shoot RII of Canada bluegrass showed interactive effects of water and temperature (Tables 3 and 4). At low temperatures, shoot and root RII of target-spotted knapweed were positive at high water input, but negative at low water input, indicating a switch of target plants from facilitation to competition (Fig. 2A and B). Shoot biomass RII of Canada bluegrass was more negative at 25 °C/22 °C and low-water inputs than at other experimental conditions, suggesting more intense competition under these conditions. Results suggest that at elevated temperatures, spotted knapweed root and shoot biomass and Canada bluegrass shoot biomass were similarly suppressed by competitors regardless of water inputs. Yellow toadflax shoot biomass RII was not affected by temperature or water treatments. Bluebunch wheatgrass shoots were more suppressed by competitors at low water and low temperatures compared to high water and elevated temperatures, respectively, while roots were more suppressed at low temperatures only (Table 3, Figs. 3A and B and 4A and B). However, yellow toadflax roots were more suppressed by competitors at high temperatures and high water (Table 4, Figs. 3A and Band 4A and B). Shoot biomass RII of all four species did not respond to the interaction of competitor identity and water or temperature, or to the interaction of all three variables (Table 3). Root biomass RII was not significantly affected by the three-way interaction, or the interaction between competitor identity and water for any of the plant species (Table 4).

**Table 3.** Three-way ANOVA table testing the relationships between water, temperature and competitor identity on relative interaction index (RII) calculated using shoot biomass of two forbs (spotted knapweed: *C. stoebe* and yellow toadflax: *L. vulgaris*) and two grasses (Canada bluegrass: *P. compressa* and bluebunch wheatgrass: *P. spicata*) commonly found in interior British Columbia. Values in bold are significant at *P* < 0.05.

	*Centaurea stoebe*			Linaria vulgaris			Poa compressa			Pseudoroegneria spicata		
	df	*F*	*P*	df	*F*	*P*	df	*F*	*P*	df	*F*	*P*
Temperature	**1, 186**	**7.13**	**0.01**	1, 161	0.32	0.57	1, 186	3.33	0.07	**1, 189**	**11.81**	**< 0.01**
Water	**1, 186**	**33.81**	**< 0.01**	1, 162	3.89	0.05	**1, 186**	**20.05**	**< 0.01**	**1, 189**	**6.29**	**0.01**
Competitor identity	**3, 186**	**11.82**	**< 0.01**	3, 143	1.34	0.26	**3, 151**	**5.49**	**< 0.01**	**3, 189**	**5.75**	**< 0.01**
Temperature × water	**1, 186**	**7.32**	**0.01**	1, 162	2.93	0.09	**1, 186**	**14.54**	**< 0.01**	1, 189	3.39	0.07
Temperature × competitor identity	3, 186	0.46	0.71	3, 143	1.56	0.20	3, 151	0.25	0.86	3, 189	0.49	0.69
Water × competitor identity	3, 186	0.50	0.68	3, 143	0.49	0.69	3, 151	0.72	0.54	3, 189	0.43	0.73
Temperature × water × competitor identity	3, 186	1.46	0.23	3, 143	1.56	0.20	3, 151	0.31	0.82	3, 189	0.24	0.87

**Table 4.** Three-way ANOVA table testing the relationships between water, temperature and competitor identity on relative interaction index (RII) calculated using root biomass of two forbs (spotted knapweed: *C. stoebe* and yellow toadflax: *L. vulgaris*) and two (Canada bluegrass: *P. compressa* and bluebunch wheatgrass: *P. spicata*) commonly found in interior British Columbia. Values in bold are significant at *P* < 0.05.

	*Centaurea stoebe*			Linaria vulgaris			Poa compressa			Pseudogeneria spicata		
	df	*F*	*P*	df	*F*	*P*	df	*F*	*P*	df	*F*	*P*
Temperature	**1, 186**	**26.06**	**< 0.01**	**1, 158**	**5.15**	**0.02**	1, 159	3.58	0.06	**1, 164**	**16.75**	**< 0.01**
Water	**1, 186**	**11.10**	**< 0.01**	**1, 158**	**11.78**	**< 0.01**	**1, 159**	**15.92**	**< 0.01**	1, 164	0.00	0.99
Competitor identity	**3, 186**	**7.23**	**< 0.01**	3, 144	2.11	0.10	3, 149	0.20	0.90	3, 154	0.75	0.53
Temperature × water	**1, 186**	**14.03**	**< 0.01**	1, 158	1.11	0.29	1, 159	1.45	0.23	1, 164	3.65	0.06
Temperature × competitor identity	3, 186	0.84	0.32	**3, 144**	**3.91**	**0.01**	3, 149	0.23	0.87	3, 154	0.39	0.76
Water × competitor identity	3, 186	0.38	0.74	3, 144	1.22	0.31	3, 149	0.38	0.77	3, 154	0.85	0.47
Temperature × water × competitor identity	3, 186	1.30	0.34	3, 144	0.89	0.45	3, 149	0.54	0.66	3, 154	0.40	0.76

## Discussion

We found mixed implications of simulated climate change on the plants examined in our study. Response to changing conditions was dependent on the individual plants rather than their status as forage grasses or exotic forbs. Reduced water input and higher temperatures, respectively, intensified and weakened competitive effects of other plants on the native forage bluebunch wheatgrass but had the opposite effects on the roots of the exotic forb, yellow toadflax. Spotted knapweed and Canada bluegrass were also more suppressed by reduced water, but only at low temperatures.

The patterns in our study for overall RII do not completely support our predictions that climate change will cause exotic species to competitively outperform species already present in the community. Elevated temperatures, at high-water inputs, allowed exotic spotted knapweed to switch from facilitative to competitive interactions and strengthened the competitive effect of other species on yellow toadflax. On the other hand, warming reduced competitive stress on bluebunch wheatgrass, and on Canada bluegrass at low water, unexpectedly. In a review of the impacts of climate change in alpine environments, Anthelme *et al*. (2014) found that warming never increased facilitation and resulted in reduced facilitation in 53 % of studies. They suggest that in these environments, facilitation is supported due to stress generated by extreme cold temperatures, in line with the predictions of the stress gradient hypothesis (Maestre *et al*. 2009; Ploughe *et al*. 2019). Due to phenotypic plasticity, invasive plants are expected to adapt better to climatic warming than native plants (Liu *et al*. 2017). Our results here suggest that for the plant species in this study, stress from warming will be more favourable for both native and exotic grasses than exotic forbs, contrary to our expectations.

Water input had contrasting effects on the exotic forbs in this study. At reduced water input, the effects of competitors on yellow toadflax roots weakened, as predicted, while spotted knapweed unexpectedly transitioned from facilitation to competition. Reduced water also resulted in stronger competitor effects on the shoots of both grasses, although only at higher temperatures for Canada bluegrass. Reductions in water have been shown to have positive, neutral, and negative effects on competitive interactions in invasive plants. Price *et al*. (2011) showed that reduced water inputs favoured an invasive forb rather than a native grass. In Maron and Marler (2008), water addition had no influence on competitive interactions between exotic plants (spotted knapweed and Dalmatian toadflax), and native plants. Sheley and James (2014) found that invasive forbs were more suppressed than native grasses when water is low. They suggest that this may happen in low-resource scenarios where thinner tissue construction requirements of invaders allow for faster growth but decreases tissue life span and reduces the ability to maintain physiological function. The performance of yellow toadflax may be also be linked to its ability to develop fast-growing creeping roots that facilitate dense growth (McClay and De Clerck-Floate 2001; Jacobs and Sing 2006). Root biomass of yellow toadflax was highest, although not statistically significant, under competition and low water. This, coupled with significant reduction of spotted knapweed’s biomass, and mild reductions in grass biomass at low water, may explain yellow toadflax’s success. In a meta-analysis, Kiær *et al*. (2013) found that root competition was generally stronger than shoot competition for smaller competitors at low resource levels. It appears then that reduced water will adversely affect exotic spotted knapweed and forage grasses, while exotic yellow toadflax will be less competitively suppressed.

Species-specific responses in our study are likely due to individual plant characteristics. Lüscher *et al*. (2022) suggest that plants may use different strategies to tolerate periods of drought. Some plants resist drought by maintaining biomass production during dry periods, while others survive drought by ceasing growth during dry periods but recover when water is more abundant. Sperber (2001) found that spotted knapweed can produce deep roots to gain access to water, leading to increased water stress tolerance. Competition with the other plants that do not produce deep roots may decrease the advantage of investment into root production, resulting in spotted knapweed being outcompeted. The ability of yellow toadflax to produce many roots quickly (Holdorf 2003; Jacobs and Sing 2006), in addition to grass shoot plasticity (Fraser *et al*. 2009), may drive the responses observed in this study.

Taken together, our results indicate that to effectively manage exotic species in light of climate change, biotic interactions between native and exotic species should be considered (Montoya and Raffaelli 2010). We used pair-wise interactions between species, to explore biotic interactions, however, response to climatic drivers in experimental studies may depend on the selected species, or unobserved conditions (McCluney *et al*. 2012). Experimental outcomes of interactions between target and competitor plants may also differ between seedling and adults. In addition, responses observed under controlled conditions may vary from the field environment. Our findings solidify the necessity of studying climate change impacts on interactions between species and specify the need for studying a suite of species.

## Supporting Information

The following additional information is available in the online version of this article –

**Table S1.** Post hoc tests showing the main effects of water on shoot biomass of yellow toadflax.

**Table S2.** Post hoc tests showing the main effects of temperature on shoot and root biomass of bluebunch wheatgrass.

**Table S3.** Post hoc tests showing the main effects of competition on shoot biomass of bluebunch wheatgrass.

**Table S4.** Post hoc tests showing the main effects of competition on shoot biomass of Canada bluegrass.

plad017_suppl_Supplementary_Material

## Data Availability

Data used for this paper is available at: https://doi.org/10.6084/m9.figshare.22351297.v2.

## Notes

**Populations & Communities. Guest editor:** Jean Burns
