# Supplementary material for: Winner and losers: examining biotic interactions in forbs and grasses in response to changes in water and temperature in a semi-arid grassland
Source: AoB Plants. 2023 Apr 24;15(3):plad017. doi: 10.1093/aobpla/plad017 (PMC10184435; doi:10.1093/aobpla/plad017)
Supplement: plad017_suppl_Supplementary_Material [file plad017_suppl_supplementary_material.docx]

SUPPORTING INFORMATION

Table 1

Post hoc tests showing the main effects of water on shoot biomass of yellow toadflax.

| Water | Value | SE | Lower CL | Upper CL |  |
| --- | --- | --- | --- | --- | --- |
| High | 0.056 | 0.012 | 0.023 | 0.108 | a |
| Low | 0.082 | 0.018 | 0.043 | 0.157 | b |

Table 2

Post hoc tests showing the main effects of temperature on shoot and root biomass of bluebunch wheatgrass*.*

| Shoot biomass | | | | | |
| --- | --- | --- | --- | --- | --- |
| Temperature | Value | SE | Lower CL | Upper CL |  |
| Low | 0.543 | 0.055 | 0.440 | 0.670 | a |
| High | 0.384 | 0.039 | 0.311 | 0.742 | b |
| Root biomass | | | | | |
| Low | 0.288 | 0.046 | 0.206 | 0.403 | a |
| High | 0.177 | 0.028 | 0.127 | 0.248 | b |

Table 3

Post hoc tests showing the main effects of competition on shoot biomass of bluebunch wheatgrass*.*

| Competition | Value | SE | Lower CL | Upper CL |  |
| --- | --- | --- | --- | --- | --- |
| Competitor present | 0.385 | 0.025 | 0.305 | 0.486 | a |
| Competitor absent | 0.541 | 0.061 | 0.428 | 0.683 | b |

Table 4

Post hoc tests showing the main effects of competition on shoot biomass of Canada bluegrass.

| Competition | Value | SE | Lower CL | Upper CL |  |
| --- | --- | --- | --- | --- | --- |
| Competitor present | 0.663 | 0.083 | 0.392 | 1.120 | a |
| Competitor absent | 0.898 | 0.128 | 0.586 | 1.380 | b |
